# Seasonality and heterogeneity of malaria transmission determine success of interventions in high-endemic settings: a modeling study

**DOI:** 10.1186/s12879-018-3319-y

**Published:** 2018-08-22

**Authors:** Prashanth Selvaraj, Edward A. Wenger, Jaline Gerardin

**Affiliations:** Institute for Disease Modeling, Bellevue, WA USA

**Keywords:** Malaria, Seasonality, Heterogeneity Mathematical modeling

## Abstract

**Background:**

Malaria transmission is both seasonal and heterogeneous, and mathematical models that seek to predict the effects of possible intervention strategies should accurately capture realistic seasonality of vector abundance, seasonal dynamics of within-host effects, and heterogeneity of exposure, which may also vary seasonally.

**Methods:**

Prevalence, incidence, asexual parasite and gametocyte densities, and infectiousness measurements from eight study sites in sub-Saharan Africa were used to calibrate an individual-based model with innate and adaptive immunity. Data from the Garki Project was used to fit exposure rates and parasite densities with month-resolution. A model capturing Garki seasonality and seasonal heterogeneity of exposure was used as a framework for characterizing the infectious reservoir of malaria, testing optimal timing of indoor residual spraying, and comparing four possible mass drug campaign implementations for malaria control.

**Results:**

Seasonality as observed in Garki sites is neither sinusoidal nor box-like, and substantial heterogeneity in exposure arises from dry-season biting. Individuals with dry-season exposure likely account for the bulk of the infectious reservoir during the dry season even when they are a minority in the overall population. Spray campaigns offer the most benefit in prevalence reduction when implemented just prior to peak vector abundance, which may occur as late as a couple months into the wet season, and targeting spraying to homes of individuals with dry-season exposure can be particularly effective. Expanding seasonal malaria chemoprevention programs to cover older children is predicted to increase the number of cases averted per treatment and is therefore recommended for settings of seasonal and intense transmission.

**Conclusions:**

Accounting for heterogeneity and seasonality in malaria transmission is critical for understanding transmission dynamics and predicting optimal timing and targeting of control and elimination interventions.

**Electronic supplementary material:**

The online version of this article (10.1186/s12879-018-3319-y) contains supplementary material, which is available to authorized users.

## Background

Malaria prevalence has decreased worldwide over the last two decades, yet the Sahelian region of sub-Saharan Africa continues to experience considerable burden [1]. Transmission in the Sahel is seasonal and intense [2–4], presenting a challenge to control programs and raising the question of whether and how malaria elimination could be achieved in this region.

When transmission is extremely seasonal, the human parasite reservoir declines through the dry season and reaches its nadir at the beginning of the wet season as vector numbers begin to rise [5]. This pattern presents opportunities for effective control and potential elimination. Because transmission is so seasonal, interventions such as seasonal malaria chemoprevention (SMC) are recommended to be distributed to children prior to and during the peak transmission season to reduce clinical episodes [6]. For elimination purposes, the period immediately prior to the wet season may represent a vulnerable point in seasonal settings where cleverly designed intervention programs may have an outsized impact and potentially interrupt transmission. To properly design such a program, it is first necessary to understand seasonal exposure at a level of realism beyond simple assumptions of seasonality as sine or box shapes.

The Garki Project of the 1970s was a comprehensive study in northern Nigeria that paired longitudinal entomology and parasitology measurements with aggressive vector control and drug-based interventions to investigate the feasibility of malaria elimination in the Sahel [2]. While malaria transmission was ultimately reestablished in the study sites following cessation of interventions, data from this study continues to prove an invaluable source of insight into the links between exposure, infection, and acquisition of immunity. In most villages, the Garki study found no evidence of infectious bites in the small number of vectors found during the dry season. However, concurrent parasitology measurements in children in the same villages found high-density infections in the hotter months of the dry season following several previous low or negative measurements, suggesting some biting occurs during the dry season, likely at rates low enough to evade entomological surveys. Under the right modeling framework, parasitological data should be able to supplement entomological data to inform our understanding of local seasonality and heterogeneity of exposure.

Interest in indoor residual spraying (IRS) has been rekindled in recent years, and it is increasingly considered to be a key component of integrated malaria management [7]. When well-implemented, IRS is an efficient intervention to reduce vector density and lifespan, especially when vectors in a region are highly endophilic [8], as is common in regions across the Sahel [9, 10]. IRS has proven a powerful tool in reducing indoor biting in Sahelian settings even when vector populations are dominated by exophilic species [11]. However, the cost of IRS can be prohibitive for resource-limited programs. Understanding how IRS timing affects campaign outcomes and whether targeting IRS to certain households can further increase impact would help programs optimize deployment of this intervention.

Drug campaigns reduce morbidity through prophylaxis and decreasing human transmission to mosquitoes. Following successful trials of SMC in the Sahel using full-treatment regimens of sulfadoxine-pyrimethamine plus amodiaquine (SP-AQ) [12, 13], WHO recommended the use of SMC to reduce burden in children between 3 and 59 months in highly seasonal malaria settings in sub-Saharan Africa in 2012 [14]. Since then, the use of SMC in the region has increased consistently with three countries adopting the program in 2013, nine in 2015, and eleven in 2016 [15]. Some programs are considering expanding SMC to include children up to 120 months of age as long as the expansion is cost-effective [16] and safe [17]. However, accessibility issues during the rainy season pose substantial challenges to implementing SMC [18], and distributing mass drug administration (MDA) or mass test-and-treat (MTAT) instead, but with fewer rounds, may result in similar performance. Interest in MDA and MTAT campaigns has been renewed recently in the context of malaria elimination. MDA campaigns, where individuals of all ages receive presumptive treatment, have been recommended by the WHO Malaria Policy Advisory Committee for interrupting transmission in low transmission settings or to reduce morbidity and mortality under exceptional circumstances [19]. However, the committee withheld guidance for the use of these drug campaigns in moderate or high transmission settings pending further evidence of their effectiveness, and recommended the development of a research consortium to oversee studies that would inform future recommendations.

Where testing interventions in the field is time-consuming and expensive, mathematical modeling can provide insight into which factors affect intervention outcome and potential pitfalls that should be avoided if possible [20–24]. To predict the effect of intervention outcomes in extremely seasonal settings such as the Sahel, it is necessary to understand immunity at the seasonal timescale as well as the degree and nature of heterogeneity in dry-season exposure. This work uses data from the Garki project to calibrate a mathematical model of malaria transmission at seasonal resolution. The model is then used to predict the optimal times at which to deploy vector control and drug-based interventions, compare various possible implementations of SMC, and test whether targeting high-risk groups with higher intervention coverage is a promising strategy for malaria control in high-endemic areas.

## Methods

### Simulation framework

Simulations were carried out with EMOD v2.11 [25], an agent-based mechanistic model of malaria transmission with vector life cycle [26] and within-host parasite and immune dynamics [27, 28]. Both asexual parasite density and gameteocyte densities by stage are tracked within each host. The presence of asexual parasites during the blood stage of a malaria infection stimulates innate and adaptive immunity within the host. Innate immunity produces cytokines and fever that limit maximum parasite density. Adaptive immunity to asexual parasites is modeled using three types of antigens: *Plasmodium falciparum* erythrocyte membrane protein 1 (PfEMP1) variants, merozoite surface proteins (MSP), and minor epitope variants. Gametocytes differentiate from asexual parasites and mature in five stages over 10 days, and a fraction of gametocytes is lost at each stage due to clearance by the host. Mosquito blood meals result in density-dependent uptake of mature stage gametocytes, and survival of gametocytes within a mosquito is dependent on both human and mosquito immune factors.

### Calibration methodology

Calibrations followed the approach in [5, 29]. The within-host portion of the model was calibrated using incidence and prevalence data from nine study sites in sub-Saharan Africa: Namawala in Tanzania [30] (prevalence); Matsari, Rafin Marke, and Sugungum in Nigeria [2] (prevalence); Dielmo and Ndiop in Senegal [31] (incidence); and Dapelogo and Laye in Burkina Faso [32] (prevalence). Infectiousness of humans to mosquitoes was calibrated to data from the Burkina Faso sites. Prevalence data from the Nigerian sites were stratified by age, month, and asexual parasite density. Asexual and gametocyte densities from the Burkina Faso sites were stratified by age and season and were measured by molecular methods, while prevalence and density measurements in other sites were obtained by microscopy. In the Dielmo and Ndiop sites, 30% of symptomatic cases received curative treatment in the model [29]; other study sites were modeled without case management as treatment rates were believed to be low.

The model parameters under calibration included the maximum number of infections that an individual can have simultaneously, number of PfEMP1 variants in the overall parasite population, switching rate between PfEMP1 variants, number of MSP variants in the overall parasite population, fraction of merozoites inhibited from invading new erythrocytes when MSP1-specific antibody level is at maximum, number of minor epitope variants in the overall parasite population, kill rate of infected red blood cells due to antibody response to minor epitopes, fraction of infected red blood cells producing gametocytes, and survival rate of gametocytes as they progress through stages of maturation. The likelihoods of each parameter set were calculated as described in [5, 29] using a Dirichlet-multinomial distribution to compare simulation data with field data.

**Fig. 1:**
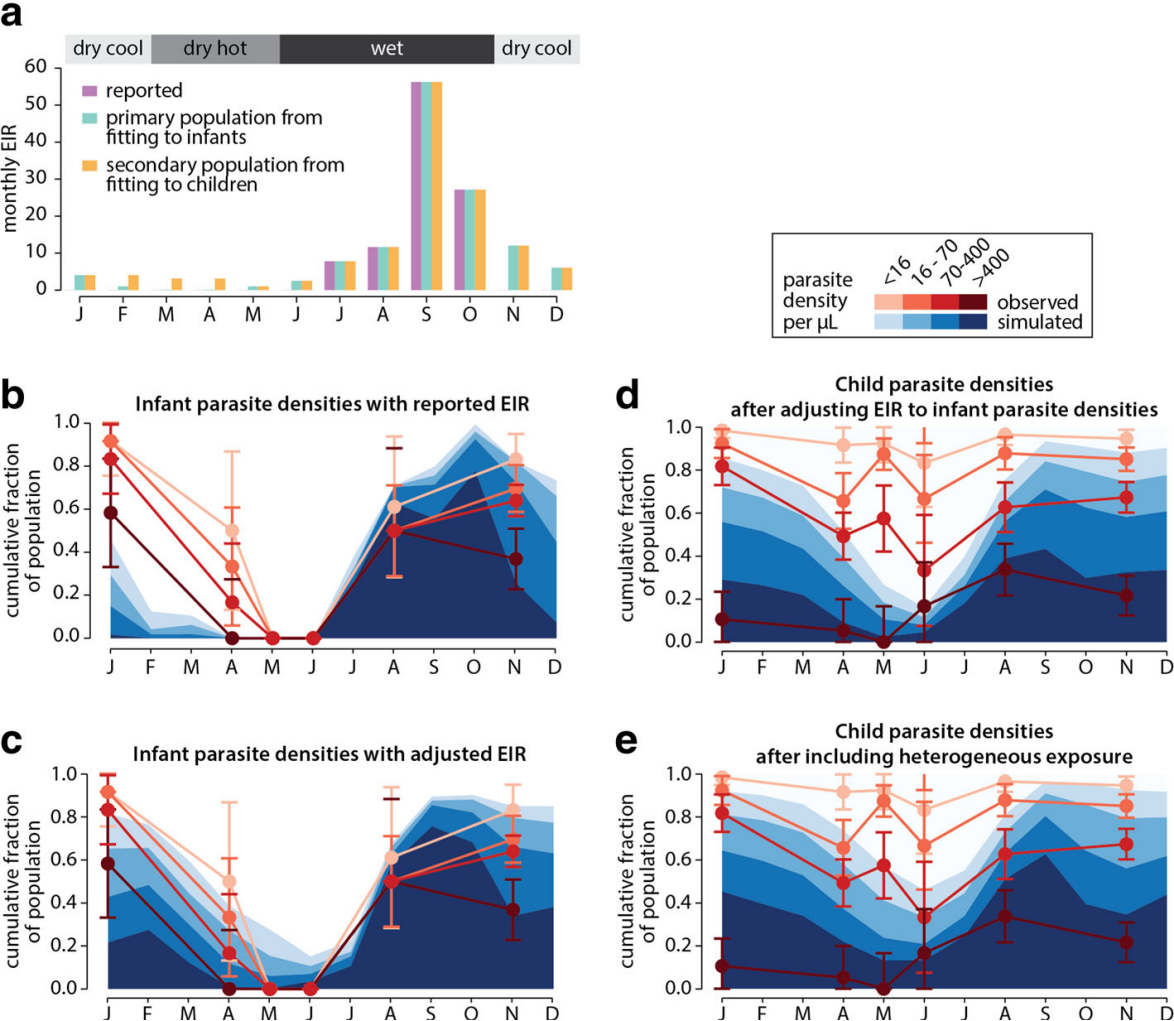
Inferring EIR from infant and child conversion rates. **a** Reported EIR from Matsari site (purple), EIR inferred from fitting to infant asexual parasite densities (green), and EIR of a high-biting population inferred from fitting to child asexual parasite densities (yellow). **b** Infant parasite densities observed (red) and simulated (blue) under reported EIR. **c** Infant parasite densities observed (red) and simulated (blue) after adjusting dry-season EIR. **d** Parasite densities in children between 4 and 8 years of age observed (red) and simulated (blue) with inferred EIR from adjusting to infant parasite densities. **e** Parasite densities in children between 4 and 8 years of age observed (red) and simulated (blue) after including heterogeneous dry-season biting and calibration of immune model

Calibration simulations followed a birth cohort of 1000 children over 70 years. During calibration, simulations were carried out in the absence of vectors using a forced EIR where individuals received a predetermined number of infectious bites calculated from monthly entomological data. In the Burkina Faso sites, modeled seasonality followed entomological observations, with an annual EIR of 300 in Dapelogo and 30 in Laye [33]. In the Garki sites, monthly EIR was calculated by averaging the product of human biting rate and sporozoite rate over the days of the month when data was collected [2], then multiplying this value by the number of days in the month. Parasite prevalence data from infants, stratified by density and month of observation, were used to infer monthly EIR when entomological data was not available. Monthly EIR values were adaptively tuned for a given parameter set until there was a good fit of simulation data to the reference dataset (Fig. 1). To address dry-season biting, which appeared to be minimal in infants, the population was divided into two groups: one experiencing the infant-based EIR profile, and another experiencing higher EIR during the dry-season months of March, April, and May. Dry-season EIR values and the proportion of the population in the group with dry-season biting were each varied such that simulation closely matched reference prevalence data stratified by age, month, and density in children under the age of 18. An immune calibration was then conducted, and best fit parameters from the immune calibration were used when calibrating human infectiousness to mosquitoes via membrane feeding data stratified by age, density, and study site from the Burkina Faso sites [32]. Best fit immunity and infectiousness parameters are shown in Additional file 1: Table S1.

### Uncertainty in measurement

Parasite densities in the Garki and Burkina Faso sites were collected using microscopy and QT-NASBA respectively. The density of pathogens measured by both microscopy and molecular methods is subject to errors, including but not limited to errors in the device (e.g. sensitivity, TTP exactitude), and user errors (e.g. reader technique, parallax type error) [34, 35]. Statistical inference analysis of reference data was used to provide uncertainty estimates on measured densities in the Burkina Faso sites using a Bayesian mixed model approach as described in [36]. In the Garki sites, parasite densities were estimated by counting the proportion of microscopy fields positive for 200 or 400 slide views. The detection threshold of this method corresponds to approximately 2 parasites per microlitre using 200 slide views. The probability of finding a positive field of view given a certain maximum parasite density was modeled with an exponential cumulative distribution function. Given the number of positive fields of view per 200 slide views, the mean parasite density can be calculated via

1 $$ {{\begin{aligned} \text{parasite density} = -\text{volume per field}*\log\left(1 - \frac{\text{fields positive}}{200}\right) \end{aligned}}}  $$

When there are 200 positive slide views, the calculated parasite density represents a lower bound of 410 parasites per microliter. A binomial draw was conducted on simulation data from Garki sites to account for uncertainties in microscopy readings.

### Quantifying the infectious reservoir

Analysis of the infectious reservoir was completed using forced EIR with the seasonal profile of the Matsari site and heterogeneous biting risk with 75% of individuals receiving no dry-season biting as in the green profile in Fig. 1a and 25% of individuals receiving dry-season biting as in the yellow profile in Fig. 1a. Composition of the infectious reservoir was calculated at each timepoint by measuring the probability that a mosquito feeding on an individual becomes infected, scaling by surface-area dependent biting risk [37–39], and summing these probabilities across all individuals in the simulation. Individuals were categorized according to their age, detectability of their asexual infection by various detection methods, and whether or not they were exposed to dry-season biting. Detection methods of asexual infection included rapid diagnostic test (RDT) with sensitivity of 40 parasites per *μ*L, high-sensitivity RDT (hsRDT) with sensitivity of 4 parasites per *μ*L, and PCR with sensitivity of 0.1 parasites per *μ*L.

To compare the infectious reservoir at different EIR, the Matsari EIR profile was scaled down to sample annual EIR between 0.1 and 100 while maintaining the same seasonality. Infectiousness was averaged over the year.

### Vector transmission model

To characterize the effect of timing and targeting interventions to specific subpopulations within a geographic area, malaria transmission was simulated in a highly seasonal setting with explicit inclusion of vectors and vector life cycle dynamics and the best-fit immunity and infectiousness parameters obtained from the calibration described above. The *Anopheles gambiae* complex is one of the dominant vectors in the Sahel, although the precise mix of species and behaviors shifts from north to south across the region, and there can be significant variation in species from village to village [2, 40]. In this work, the vector population was modeled as *An. gambiae* mosquitoes with 90% indoor feeding and resting rate. Monthly habitat availability was selected such that the resulting EIR profile and prevalence rates in the model were similar to those observed in the Matsari group experiencing dry-season biting (Fig. 1a). In simulations with homogeneous exposure, all individuals experienced some dry-season biting, with biting risk modulated only by age as described above. Total human population was 1000 individuals with birth and death rates of 45 per 1000 per year, resulting in about 20% of the population being under 5 years of age and 15% between 5 and 10 years of age.

In simulations with heterogeneous exposure, the proportion of the population that experiences dry-season biting was varied from 0 to 90%. The total number of bites was conserved across different proportions of the population that experiences dry-season biting by varying biting risk in each population for each month based on the ratios of low to high biting EIRs. Additional file 2: Figure S7 shows the difference in biting between the two groups for each month of the year.

To initialize population immunity prior to testing vector control and drug campaign scenarios, simulations were run for 65 years in the absence of any interventions, then an additional 5 years with case management with artemether-lumefantrine (AL), where drug pharmacokinetics and pharmacodynamics are explicitly modeled [41]. Case management rates were set at 50% for clinical malaria in children 5 and under, 30% for clinical malaria in individuals over 5, and 50% for all cases of severe malaria. All intervention scenarios also included case management at these rates.

### Interventions

Indoor residual spraying (IRS), seasonal malaria chemoprevention (SMC) in children under 5 and 10 years old, and mass drug administration (MDA) and mass test-and-treat (MTAT) in all individuals were considered for control strategies. Case management was modeled as described above. No other vector control interventions were modeled and drug and insecticide resistance were assumed to be absent.

Optimal IRS timing under homogeneous biting conditions was evaluated by sampling dates of spraying at 10-day increments over the year and calculating the percent reduction in prevalence observed one year after the spray date: (prevalence immediately prior to spraying − prevalence one year after spraying)/(prevalence immediately prior to spraying). Four possible half-lives for IRS were tested: 60 days (bendiocarb-like) [42], 100 days (propoxur-like) [42], 180 days (Actellic-like) [43], and a hypothetical chemical with a half-life of 250 days. Coverage of IRS was tested at 50% at 80%. Fifty stochastic realizations were run for each spray date, half-life, and coverage combination.

Under heterogeneous biting conditions, IRS coverage in the group with dry-season biting as well as the fraction of the population experiencing dry-season biting were sampled. IRS coverage in the group with no dry-season biting was calculated to maintain constant overall coverage:

2 $$ \textrm{LBC} = \frac{\textrm{overall coverage} - \textrm{HBF*HBC}}{1-\textrm{HBF}},  $$

where LBC is the coverage in the group experiencing lower dry-season biting, HBC is the coverage of the group experiencing high dry-season biting, and HBF is the fraction of the population experiencing high dry-season biting. Spraying time was sampled monthly and a half-life of 180 days was assumed. Overall coverage was tested at 50% and 80%, and 25 stochastic realizations were run for each combination of spray date, overall coverage, HBC, and HBF.

Optimal timing of drug campaigns under homogeneous biting conditions was evaluated by sampling the date of the first drug campaign round at 10-day increments over the year and calculating the number of clinical cases averted relative to a baseline without drug campaigns. Clinical cases were defined as malarial fevers of at least 38.5°C occurring at least 14 days since the previous fever. SMC campaigns consisted of four rounds separated by 30 days and two options, treating children 5 and younger (standard SMC) or children 10 and younger (expanded SMC). Children were presumptively treated with DHA-piperaquine (DP) during SMC regardless of infection status. MDA campaigns were simulated with 2 rounds separated by 30 days to test whether fewer rounds but treating more people per round could result in similar effectiveness as SMC. MTAT campaigns were simulated at 3 rounds separated by 60 days, as the time and labor-intensive nature of conducting both tests and treatments in the field requires longer intervals between rounds, and an extra round of tests and treatments ensured prophylaxis comparable to the MDA and SMC campaigns tested.

Individuals of all ages were eligible for MDA and received DP regardless of infection status. Under MTAT, individuals of all ages were eligible to receive an RDT with sensitivity at 40 parasites per *μ*L, and individuals who tested positive received DP. Coverages of 50% and 80% were tested for SMC, MDA and MTAT campaigns, and coverage was independent between rounds. Fifty stochastic realizations were run for each drug campaign type, date, and coverage combination.

In the heterogeneous biting scenarios, the overall coverage of MTAT and SMC campaigns was kept constant, and the coverage and fraction of the group experiencing high dry-season biting was varied with the corresponding coverage in the other group calculated using Eq. (2). The MTAT and SMC campaigns were simulated as in the homogeneous case. Optimality of timing was explored by sampling the start date of each month of the year for different coverages and fractions of the high dry-season biting group with 25 stochastic realizations for each combination of campaign start date, overall coverage, HBC, and HBF.

## Results

### Calibration of immunity and infectiousness model to monthly and seasonal measurements from the Sahel

Calibration of the immunity model to monthly parasite data requires a monthly force of infection as a model input. Monthly entomological inoculation rates (EIRs) for each Garki site can be calculated from human biting rates and sporozoite rates collected during the baseline measurement phase of the Garki project [2] (Fig. 1a, purple bars). This seasonal profile shows zero biting during the hot and cool dry-season months. However, parasite densities measured in infants, who have little to no immunity, remain high as late as January, four months after peak EIR. By April, individuals born after October account for half the infant population, yet high-density infections are still prevalent even though the entomological data would predict zero exposure for these newborns. The simulations with the reported EIR fail to capture the high-density infections observed at this time (Fig. 1b).

Tuning the EIR profile in each site to calibrate parasite prevalence in simulated infants to those from the Garki regions (Fig. 1c) results in a profile with non-zero biting throughout the year, albeit with a dramatically reduced rate in the dry season (Fig. 1a, green bars). Given the low numbers of mosquitoes collected in the dry hot months, the infant-conversion rate based EIR profile is still consistent with zero measured sporozoite positive mosquitoes. This new profile was still unable to capture high parasite densities in the dry-season months of March through May in children under the age of 8 (Fig. 1d), and increasing EIR for the entire population in the dry season resulted in overshooting parasite prevalence in the wet season. However, malaria transmission is heterogeneous [44, 45], and heterogeneous biting within the population during the dry season could account for higher densities at the population level in the dry season without overestimating wet season prevalence. The population was divided into two groups, one experiencing an EIR profile similar to the one seen in infants, and another experiencing higher EIR during the dry-season months of March, April, and May (Fig. 1a, purple bars). The relative sizes of the population fraction exposed and not exposed to dry-season biting were tuned independently for each Garki site to match dry-season parasite densities in young children (Fig. 1e). This process ultimately resulted in a much better fit to the prevalence data recorded during the study.

**Fig. 2:**
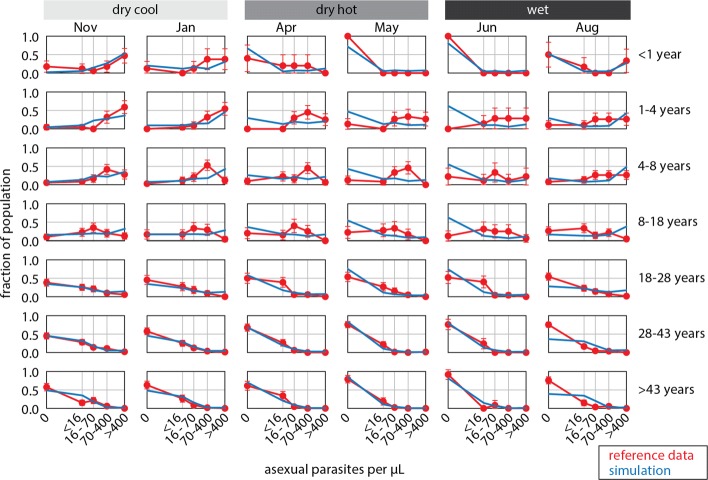
Asexual parasite density by month and age group in the Matsari site after calibrating immune parameters. Red: reference data from the Garki Project; blue: calibrated simulation

Figure 2 shows a comparison of simulation to reference data in one study site after calibrating the immune model to data from nine sub-Saharan African sites. Fits to data from other sites are shown in Additional file 1: Table S1. In Garki sites, the calibrated model shows very good agreement with data in infants and adults, but poorer fit to children, particularly in the dry-hot season between March and the end of May. This pattern of poorer fit is much less marked in the Burkina Faso sites, where the model is well able to capture parasite densities by age at all three seasonal timepoints.

Several factors could contribute to the difficulty of fitting partial immunity in the Garki sites. Our model of heterogeneity in transmission, where one subpopulation is exposed to dry-season biting and one is not, is simple and may not adequately reflect the true heterogeneity in timing of dry-season bites. In addition, the model assumes that all parasite strains are equally present, which means that in high-transmission settings like Garki, simulated young children have been exposed to nearly all strains by the time they are a few years old; in reality, it may be more likely that strains are somewhat segregated by village or season such that most exposures are from repeated strains, and exposure to a new strain will result in the higher densities observed in the Garki data. Longer duration infections may enable a portion of the population of partially-immune individuals to maintain parasite densities at intermediate levels through the dry season, although the model used in this study tends to produce longer infections than are observed in the field [46].

### Composition of the infectious reservoir of malaria in highly seasonal settings

**Fig. 3:**
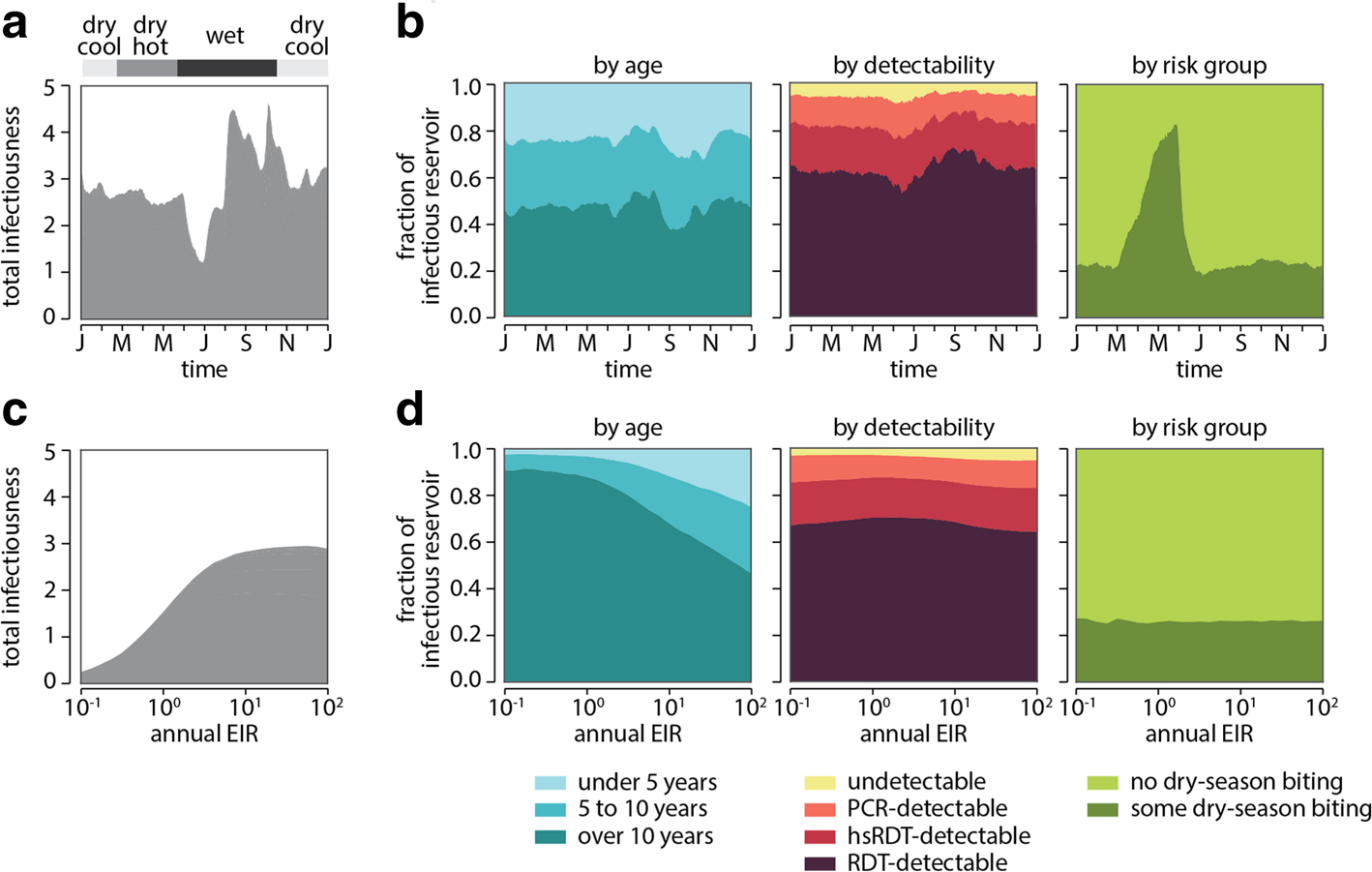
The infectious reservoir of malaria in a seasonal setting stratified by age, detectability of infection status, and susceptibility to dry-season biting. Results presented are means of 50 stochastic realizations. Annual EIR in panels A and B is 130. **a** Total human infectiousness varies through the year in a seasonal setting. **b** Contribution to the human infectious reservoir (normalized total human infectiousness) by age group, detectability, and dry-season biting risk varies through the year. **c** Annual average total human infectiousness under the same seasonality increases with EIR. **d** Contribution to the annual average human infectious reservoir by age group, detectability, and dry-season biting risk varies with EIR

The infectious reservoir of malaria varies by season and transmission intensity (Fig. 3). In highly seasonal regions such as the Sahel, the infectious reservoir peaks in size during the wet season but maintains a substantial size into the dry season and reaches its lowest point immediately prior to the next wet season (Fig. 3a).

In high-transmission areas such as the Garki region where annual EIR can reach above 100 infectious bites per person per year, adults have strong immunity to high-density parasitemia and therefore often have few gametocytes and are relatively uninfectious. In contrast, children form the majority of the infectious reservoir in all seasons (Fig. 3b). For areas with lower transmission, most of the infectious reservoir lies in adults. The work presented here assumes a demographic structure where half the population is under 15 years old, a common age structure in the Sahel, and shifting the age pyramid toward older populations as is the case in many lower-transmission areas will further increase the adult share of the infectious reservoir in those settings.

Consistent with previous studies [5, 47], individuals whose infections are RDT-detectable comprise over half the infectious reservoir. However, adjusting for measurement uncertainty results in even greater weighting of the infectious reservoir toward RDT-detectable infections, which are 65–75% of the infectious reservoir depending on transmission intensity and in high-transmission areas, between 60–80% of the infectious reservoir at any time of year and peaking during the wet season.

In a simulation where 25% of individuals are subject to dry-season biting, these individuals contribute up to 80% of the infectious reservoir during the dry season. Interventions that can target individuals with dry-season exposure at high coverage would likely exert an outsize effect on transmission.

### Timing, insecticide half-life, and targeting of high-risk populations impact effectiveness of IRS campaigns

Sustained dry-season biting by *An. gambiae* has been observed in multiple endemic, sub-Saharan settings [48, 49]. Given these feeding characteristics of *An. gambiae*, vector control interventions like IRS may offer a very effective strategy for malaria control in Sahelian settings. A model of the Matsari site of the Garki Project, where peak vector abundance is reached late in the wet season, is used to test how campaign timing and insecticide half-life impact the effect size of IRS.

**Fig. 4:**
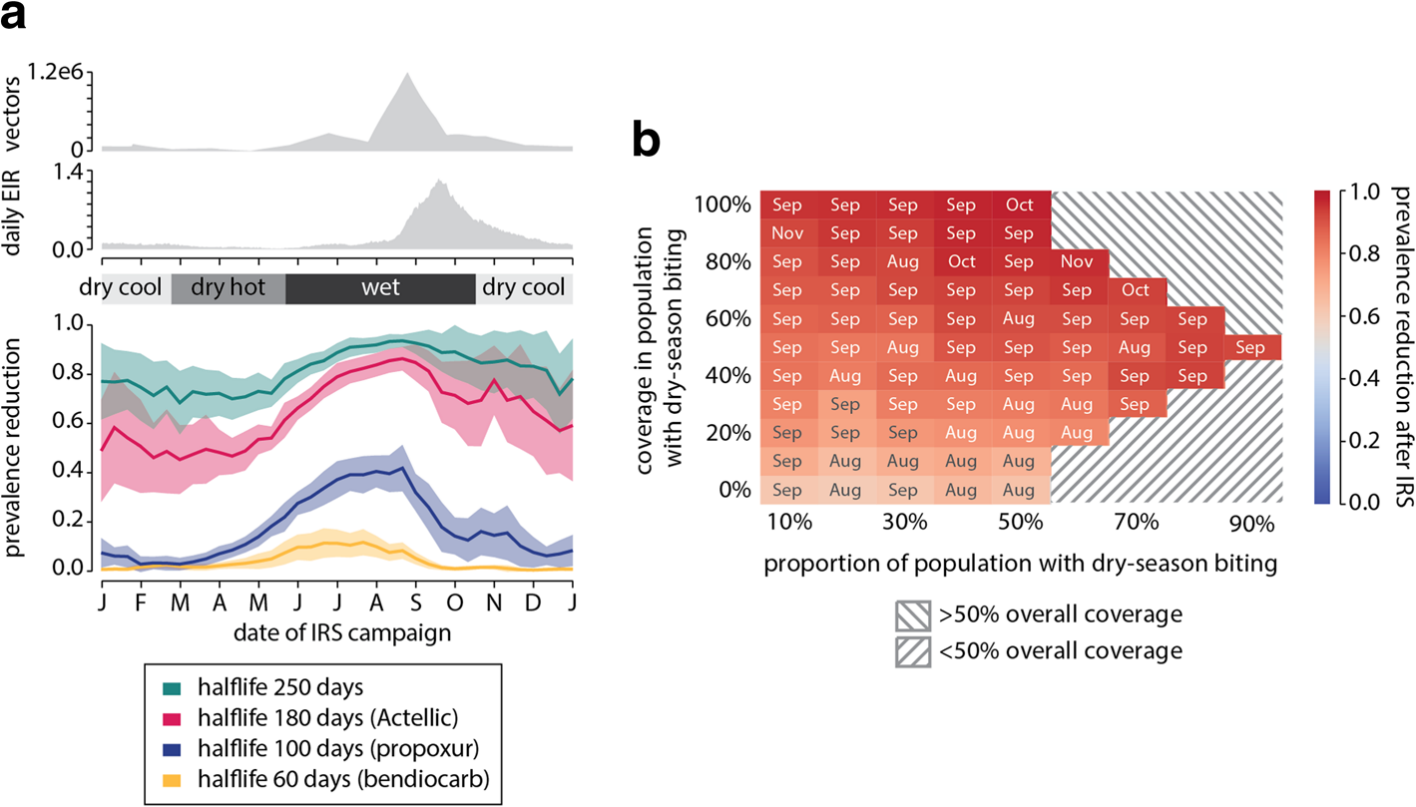
Optimal deployment of IRS. **a** Effectiveness of IRS depends on both IRS half-life and timing of spray campaigns relative to the peak biting season. Timing has the strongest impact on moderately long-lasting IRS. Top: daily count of adult vectors and EIR. Bottom: fractional reduction in prevalence one year after an IRS campaign with 50% coverage in a population with homogeneous transmission. Mean and standard deviation of 50 stochastic realizations are shown for each half-life and campaign start date. **b** The matrix of shaded boxes represents prevalence reduction in a population experiencing heterogenous dry-season biting with the coverage in population experiencing dry-season biting along the vertical axis, and the fraction of the same population in the entire population along the horizontal axis. Each box within the matrix is shaded to reflect the mean number of clinical cases averted during the year after the campaign is started for 50 stochastic realizations. The optimal start month for each campaign given the coverage and fraction of the group experiencing dry-season biting is indicated in the box. Regions shaded with gray oblique lines indicate coverage in the total population was either above or below the target 50% overall coverage for the corresponding coverage and fraction of the group with dry season biting

Campaign timing strongly impacts the reduction in prevalence observed a year after an IRS campaign (Fig. 4). In a population experiencing homogeneous biting, peak prevalence reduction is achieved when IRS is administered towards the end of August, corresponding to the peak in vector abundance, irrespective of the insecticide used (Fig. 4a). An Actellic-like chemical with a 6-month half-life, for example, results in almost 80% prevalence reduction if the campaign is started at the end of August. However, starting the same campaign in the middle of the dry season in April or May leads to only 50% reduction in prevalence. In the case of the Actellic-like chemical, the August campaign offers much more effective malaria control, with greater reduction in transmission, than a dry-season campaign. This contrast in the effectiveness of a campaign at different dates is even greater if chemicals with shorter half-lives are used. With a propoxur-like chemical with half-life of 3 months, maximum prevalence reduction of about 45% is achieved when spraying starts at the end of August during the peak wet season, while almost no prevalence reduction is observed a year after a March campaign.

IRS chemicals with intermediate half-lives show the largest difference in prevalence reduction between the worst and best campaign times. When a chemical with a very long half-life is used, effect size is large no matter when spraying is started, and when the half-life is short, effects are small irrespective of campaign date. However, despite the smaller difference in best and worst timing using these chemicals, spraying close to the peak wet season is still the best time, and spraying in the hot dry season the worst.

Maximizing the overlap between vector abundance and IRS killing strength averts the greatest amount of biting, which in turn maximizes the decrease in transmission through the wet season. When IRS is administered at the end of August, long half-lives ensure vector abundance is suppressed even through the following dry season. This in turn leads to the infectious reservoir being suppressed at least until next April, when the reservoir naturally tends to its smallest size. As a result, there are fewer infected mosquitoes during the beginning of the next wet season and prevalence is reduced even a year after the IRS campaign. Increasing IRS coverage results in fewer surviving vectors and even greater reduction in prevalence throughout the year (Additional file 2: Figure S5).

When access to campaign resources is limited and the population experiences heterogeneous biting over the course of the year, targeting of IRS to the right group ensures most effective overall control (Fig. 4b). When half the population experiences dry-season biting, completely missing the group with dry-season biting during an IRS campaign results in 60% prevalence reduction. However, targeting the group with dry-season biting at 80% coverage results in over 90% reduction in prevalence, which could put this high-endemic area within reach of elimination. In another example of how targeting the right group leads to more effective control, consider a scenario where the group experiencing dry-season biting is only 10% of the total population. With IRS coverage of 90% in this small group of people, and with coverage in the rest of the population at only 45%, a campaign with a 6-month half-life insecticide can still reduce prevalence by over 90% a year after the campaign. Carefully targeting the right group of people leads to disproportionately large effects on reducing malaria transmission and potentially brings the area to near-elimination under an operationally feasible set of interventions.

In this study, the vectors have been modeled as highly anthropophilic with mostly indoor biting. When outdoor biting is non-negligible, different vector control strategies such as attractive toxic sugar baits (ATSB) [50] will be required to be as effective in reducing prevalence as the scenario described here. Irrespective of the specific vector control tools needed, the strategy of targeting dry-season biting remains generalizable. Insecticide resistance, if present, will also affect the magnitude of IRS impact and should play a role in deciding which insecticide to deploy in an IRS campaign.

### An expanded SMC campaign is more efficient than MTAT or MDA at averting clinical cases

Using our model of seasonal transmission in northern Nigeria, we predict the number of cases averted by 4-round SMC campaigns in children under 5 (standard SMC), 4-round SMC in children under 10 (expanded SMC), 2-round mass drug administration (MDA) campaigns in all individuals, and 3-round mass test-and-treat (MTAT) campaigns that test all individuals with a rapid diagnostic test (RDT) and treat those who test positive. DHA-piperaquine (DP) was selected for the mass distributions to avoid confounding effects of SP-AQ resistance, which is prevalent in many African settings [51].

**Fig. 5:**
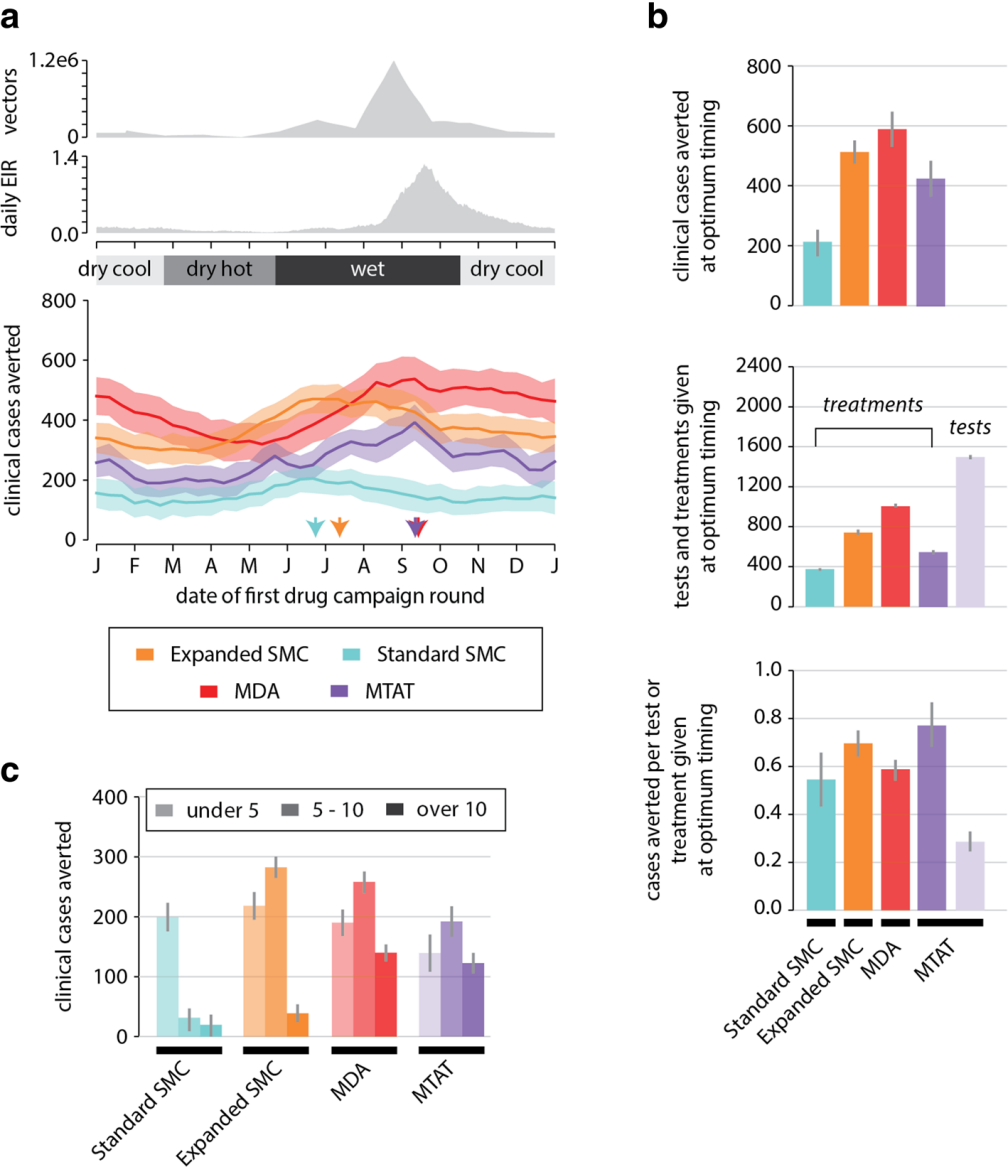
Impact of SMC, MDA, and MTAT campaigns depends on campaign timing and coverage. **a** Top: daily count of adult vectors and EIR. Bottom: clinical cases averted during the year after the first round of drugs distributed as compared to a baseline scenario with no drug campaigns. SMC, MDA and MTAT campaigns were simulated with 50% coverage. See “Methods” section for details on how drug campaigns were configured. Mean and standard deviation of 50 stochastic realizations are shown for each campaign start date. Arrows indicate the campaign start date that results in the maximum number of clinical cases averted for each campaign type. **b** Cases averted, number of tests and treatments distributed, and cases averted per test or treatment when each drug campaign type is distributed at optimum timing, at 50% coverage. **c** Cases averted by age group (under 5 years, 5 to 10 years, over 10 years) by campaign type and coverage achieved when each drug campaign type is distributed at optimum timing

For maximum effectiveness, drug campaigns must protect individuals during the peak biting months. Because SMC spans more rounds than MDA, SMC can be started earlier and also protect children during the early wet season (Fig. 5a). However, the optimum timing for MTAT is later than expected given that the simulated MTAT rounds span a period of four months. The ability to detect a large fraction of the infected population is central to the success of MTAT, and the fraction of the population testing positive by RDT increases as the rainy season gets underway. Because RDT-positive individuals are treated with parasite-clearing and prophylactic drugs, each round of MTAT makes detection of infected individuals more challenging in the subsequent round. Unless vectorial capacity is large enough that force of infection remains high, additional rounds of MTAT after the first will treat fewer individuals and have little effect on morbidity. The optimal time for distributing MTAT for burden reduction is thus late enough in the wet season that many infections are detectable, but early enough that many symptomatic cases can be averted.

Expanded SMC, MDA, and MTAT each avert more clinical cases than standard SMC (Fig. 5b) by treating age groups that are ineligible for standard SMC yet still vulnerable to clinical malaria (Fig. 5c). Even in high-transmission areas such as the one modeled here with annual EIR of 130, children older than 5 continue to experience high rates of morbidity and therefore can benefit from seasonal prophylaxis. In a simulated population of 1000 individuals with 50% coverage per round, MDA averted around 590 cases over the year following the first campaign round, expanded SMC averted around 550 cases, while the MTAT campaign averted just over 400 clinical cases and the standard SMC averted 200, which is less than half the number of cases averted by the expanded campaign.

Both standard and expanded SMC campaigns have little impact on averting clinical cases in non-targeted age groups (Fig. 5c), even when distributed at 80% coverage over 4 rounds (Additional file 2: Figure S7). Even in high-transmission settings where children form a substantial portion of the infectious reservoir, SMC campaigns reduce burden primarily through conferring personal prophylactic protection on individuals receiving treatment rather than through decreasing overall transmission by clearing the infectious reservoir.

More individuals receive treatment under MDA and expanded SMC campaigns than under MTAT and standard SMC campaigns, resulting in better performance. Taking into account the difference in number of treatments distributed in each campaign shows that SMC is highly efficient, averting 0.55 cases per treatment distributed for standard SMC and 0.70 cases per treatment for expanded SMC in a campaign with 50% coverage (Fig. 5b). The MDA campaign is slightly more efficient than the standard SMC campaign, averting 0.59 cases per treatment. MDA’s efficiency benefits from including older children but loses out in the adult population because of lower rates of morbidity in this group. In contrast, MTAT at 50% coverage averts 0.75 cases per treatment, the highest of the four drug campaigns, but only 0.28 cases per test, making MTAT almost certainly less cost-effective than SMC or MDA.

At 80% coverage, MDA and MTAT avert about as many cases per treatment given as they did at 50% coverage, while both the standard and expanded SMC campaigns avert slightly fewer cases per treatment handed out. Given uncorrelated coverage between rounds, a child may require fewer than 4 rounds per year to receive good prophylaxis, particularly if the child is old enough to have some partial immunity to disease. To ensure maximal prophylactic protection during the peak wet season, a campaign with fewer rounds would require starting later into the wet season. At higher coverage and a later start date, it is very likely most children will receive the minimum coverage for personal prophylaxis through the wet season in the first rounds, and fewer than 4 rounds are required to maximize the efficiency of burden reduction. However, the marginal utility of the fourth round also depends on the shape of local seasonality, which may not be as tightly peaked as in the village simulated here.

**Fig. 6:**
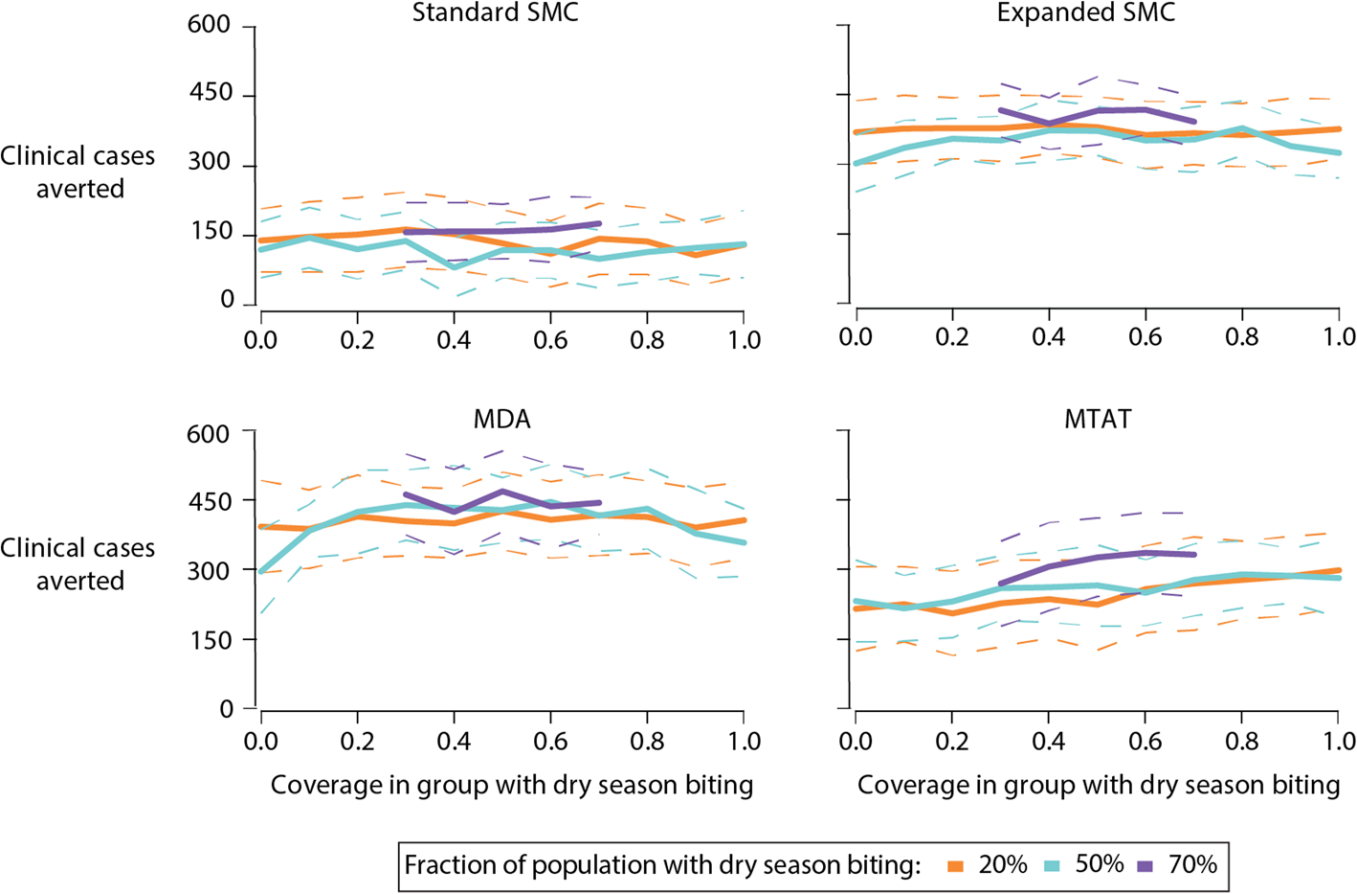
Targeting SMC, MTAT or MDA campaigns to the subpopulation experiencing dry-season biting does not increase the number of clinical cases averted in the entire population. Each campaign was started at the optimal time shown in Fig. 5a. Coverage in the group experiencing dry-season biting was varied while adjusting coverage in the remaining population such that overall coverage in each campaign round was 50%. Solid and dotted lines indicate mean and standard deviation of 50 stochastic realizations respectively

Preferentially targeting the subpopulation that experiences higher biting in the dry season during a drug campaign does not increase the total number of clinical cases averted (Fig. 6). Since all drug campaigns were timed to be distributed at or prior to the peak wet season to maximize the number of clinical cases averted, the prophylactic period does not extend into the hot dry season when there is heterogeneity in biting. At optimal timing and constant overall coverage of 50% in a population of 1000 people, MDA averts 425 clinical cases over the year following the start of the campaign irrespective of what fraction of the population experiences dry season biting or the coverage of MDA within that group. Similarly, the standard SMC, expanded SMC, and MTAT campaigns avert around 150, 400, and 225 cases, respectively.

## Discussion

In endemic regions, malaria transmission is often seasonal [52] and heterogeneous [53]. We present a model of malaria transmission that captures seasonal-timescale immune dynamics as well as seasonality and heterogeneity of exposure observed in the Sahel. The model is used to predict how vector control and drug-based intervention strategies can harness an area’s seasonality and heterogeneity to maximize impact.

EIR, an entomological quantity calculated by multiplying sporozoite rate and human biting rate, is a gold-standard measurement of transmission intensity. Entomological data is notoriously challenging to collect, as accurate measurement of sporozoite rate requires collecting and processing thousands of mosquitoes, and spatial and temporal variation in vector counts can vary hugely even across small scales [54, 55]. However, understanding seasonality and heterogeneity in biting is critical to building good models and understanding why interventions fail or succeed. When entomological data is limited, infant conversion rates and the prevalence of higher-density infections can be used to indicate recent exposure. In this study, we have leveraged a model that includes acquired immunity, and have used parasite densities to supplement entomological measurements to gain a clearer picture of seasonality despite having very limited entomological data during certain parts of the year.

When predicting intervention impact, seasonality is sometimes relegated to secondary importance compared with other operational considerations such as choice of drug or insecticide [56, 57]. However, specific features of local seasonality can greatly affect the effect size of common interventions, and modeling a simplistic seasonality such as a sine or box shape does not always lead to correct conclusions. In the Garki region examined in this work, the rainy season begins with a slow rise in vector numbers for several months before a sharp increase in August and September. For the greatest burden reduction, it is imperative that drug campaigns protect children through these peak transmission months, which requires beginning campaign rounds a month or two into the wet season rather than prior to the wet season, or even delaying later into the wet season if fewer rounds are to be given.

In Senegal, an expanded SMC campaign was well tolerated by older children and even contributed to reducing transmission in the region [16]. In higher-transmission regions like Nigeria and Burkina Faso, children experience the bulk of total burden, and we predict that an expanded SMC campaign would greatly reduce morbidity. While our model predicts a higher number of cases averted per treatment in a 4-round expanded SMC compared with a 2-round MDA, true relative impact per intervention of these two campaigns will include other operational costs that may well mean that a 2-round MDA has greater impact per intervention.

In this study, coverage in each round of the drug campaigns was assumed to be independent, which increases the access to at least one round for most people in the population [23]. However, it is more likely that the same people are difficult to access or non-participatory from round to round, which would lead to a decreased impact of drug based interventions compared with model predictions.

IRS is best timed when its most efficacious period overlaps with peak transmission months. If the insecticide is very long-lasting, IRS will be effective during peak transmission no matter when it was applied; however, for insecticides with half-life shorter than a year, timing of spray campaigns can still greatly affect this intervention’s impact [58]. When multiple vector species with different seasonal profiles are present, it becomes less clear when to distribute spray campaigns unless rigorous entomology supplies species-level data on human biting rates and insecticide resistance. In Zambia, where *An. funestus* dominates in the the dry season and *An. gambiae s.s.* in the wet season, IRS was ineffective because of high resistance to pyrethroids despite these species being predominantly endophilic [59]. IRS with non-pyrethroid chemicals such as bendiocarb or pirimiphos-methyl has been effective against a largely exophilic, pyrethroid-resistant vector when timed for maximal public health impact just prior to the peak wet season [60]. In some contexts, complementary tools such as deterrent IRS and toxic ITNs and ATSBs may be required for longer lasting transmission reduction [11, 58].

When very high coverage cannot be achieved, as is the case in resource-limited settings, targeted control offers an alternative strategy for maximizing impact of vector control. Rather than trying to spray every house in a small area, programs should consider expanding the geographic area receiving IRS but targeting vector control to high-risk households. While identifying those households may not be straightforward, information about local hydrology, heterogeneity in seasonality of clinical incidence, and seasonal migration trends can help inform which individuals are likely to experience dry-season biting.

Surveillance is acknowledged to be a pillar of successful malaria elimination programs. This work highlights the role of good surveillance and local knowledge in maximizing the impact of malaria control even in high-transmission settings, especially when resources are limited. When transmission is extremely seasonal, as in the high-burden Sahelian region of sub-Saharan Africa, individuals with higher biting risk in the dry season keep the parasite reservoir alive until the next wet season. Identifying who is at risk throughout the year is critical to understanding how transmission is sustained through the dry season or reseeded at the start of the wet season each year. As malaria burden decreases, this understanding will be central to designing effective and efficient strategies for continued control and possibly elimination.

## Conclusions

An agent-based model of malaria that includes innate and adaptive immunity was calibrated to seasonal asexual parasite and gametocyte prevalence stratified by age and density, capturing variation in within-year transmission dynamics. Where entomological data is unavailable, seasonal measurements of parasite densities in multiple age groups can be used to determine seasonality of malaria exposure.

The composition of the infectious reservoir varies seasonally in regions like the Sahel, peaking in the wet season but maintaining a considerable size throughout the year. When biting is heterogeneous during the dry season, a small fraction of people can account for most of the infectious reservoir in those months, presenting an opportunity for well-targeted interventions to have disproportionately large effects.

Impact of both vector control and drug-based interventions depends on their timing relative to peak transmission. Expanding SMC to cover children under 10 is predicted to have greater impact per intervention in areas with high seasonal transmission. When transmission is heterogeneous, good targeting of high-risk groups with vector control can have outsize effects, and missing those individuals can severely impact the success of interventions. Understanding the shape of seasonal malaria transmission and nature of heterogeneous exposure can aid in designing more efficient interventions.

## Additional files

Additional file 1 **Table S1**: Best fit parameters from immune and infectiousness calibrations. (PDF 42 kb)

Additional file 2 **Figure S1**: Measured and inferred EIRs used for calibration in Rafin Marke and Sugungum. **Figure S2**: Comparison of reference data and calibrated simulation for Dielmo, Ndiop, Namawala, Dapelogo, and Laye study sites. **Figure S3**: Comparison of reference data and calibrated simulation for Garki study sites not shown in main text. **Figure S4**: Comparison of reference data and calibrated simulation for relationship between gametocyte density and infectiousness to mosquitoes as measured in two Burkina Faso study sites. **Figure S5**: Optimal timing of IRS campaigns with IRS at 80% coverage. **Figure S6**: Cases averted and number of treatments given under various conditions of MTAT and SMC timing at 50 and 80% campaign coverage. **Figure S7**: New infections per person per month in a population where 50% of individuals experience dry-season biting and 50% do not. (PDF 1503 kb)

## Abbreviations

AL: Arthemeter-Lumefantrine
ATSB: Attractive toxicsugar baits
DHA: Dihydroartemisinin
DP: Dihydroartemisinin-Piperaquine
EIR: Entomological innoculation rate
IRS: Indoor residual spraying
MDA: Mass drug administration
MSP: Merozoite surface protein
MTAT: Mass test and treat
PfEMP1: *Plasmodium falciparum* erythrocyte membrane protein 1
QT-NASBA: Quantitative-nucleic acid sequence based amplification
RDT: Rapid diagnostic test
SMC: Seasonal malaria chemoprevention
SPAQ: Sulfadoxine-Pyrimethamine plus amodiaquine
WHO: World health organization
