# Supplementary material for: Seasonality and heterogeneity of malaria transmission determine success of interventions in high-endemic settings: a modeling study
Source: BMC Infect Dis. 2018 Aug 22;18:413. doi: 10.1186/s12879-018-3319-y (PMC6104018; doi:10.1186/s12879-018-3319-y)
Supplement: Supplementary file 1 — Table S1: Best fit parameters from immune and infectiousness calibrations. (PDF 42 kb) [file 12879_2018_3319_MOESM1_ESM.pdf]

| Parameter | Value |
| --- | --- |
| Antigen switch rate | $7.645 \times 10^{-10}$ |
| Antigenicity factor (nonspecific) | 0.4151 |
| Gametocyte production rate | 0.0615 |
| Gametocyte mosquito survival rate | 0.002 |
| Gametocyte stage survival rate | 0.5886 |
| Falciparum MSP variants | 32 |
| Falciparum nonspecific Types | 76 |
| MSP merozoite kill fraction | 0.5117 |
| Max individual infections | 3 |

Table 1: Best fit parameters from immune and infectiousness calibrations
