# Supplementary material for: Seasonality and heterogeneity of malaria transmission determine success of interventions in high-endemic settings: a modeling study
Source: BMC Infect Dis. 2018 Aug 22;18:413. doi: 10.1186/s12879-018-3319-y (PMC6104018; doi:10.1186/s12879-018-3319-y)
Supplement: Supplementary file 2 — Figure S1: Measured and inferred EIRs used for calibration in Rafin Marke and Sugungum. Figure S2: Comparison of reference data and calibrated simulation for Dielmo, Ndiop, Namawala, Dapelogo, and Laye study sites. Figure S3: Comparison of reference data and calibrated simulation for Garki study sites not shown in main text. Figure S4: Comparison of reference data and calibrated simulation for relationship between gametocyte density and infectiousness to mosquitoes as measured in two Burkina Faso study sites. Figure S5: Optimal timing of IRS campaigns with IRS at 80% coverage. Figure S6: Cases averted and number of treatments given under various conditions of MTAT and SMC timing at 50 and 80% campaign coverage. Figure S7: New infections per person per month in a population where 50% of individuals experience dry-season biting and 50% do not. (PDF 1503 kb) [file 12879_2018_3319_MOESM2_ESM.pdf]

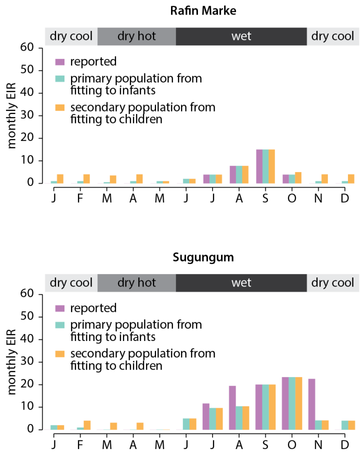

Figure 1: Measured and inferred EIRs used for calibration in Rafin Marke and Sugungum. Het pop split for Rafin Marke and Sugungum?

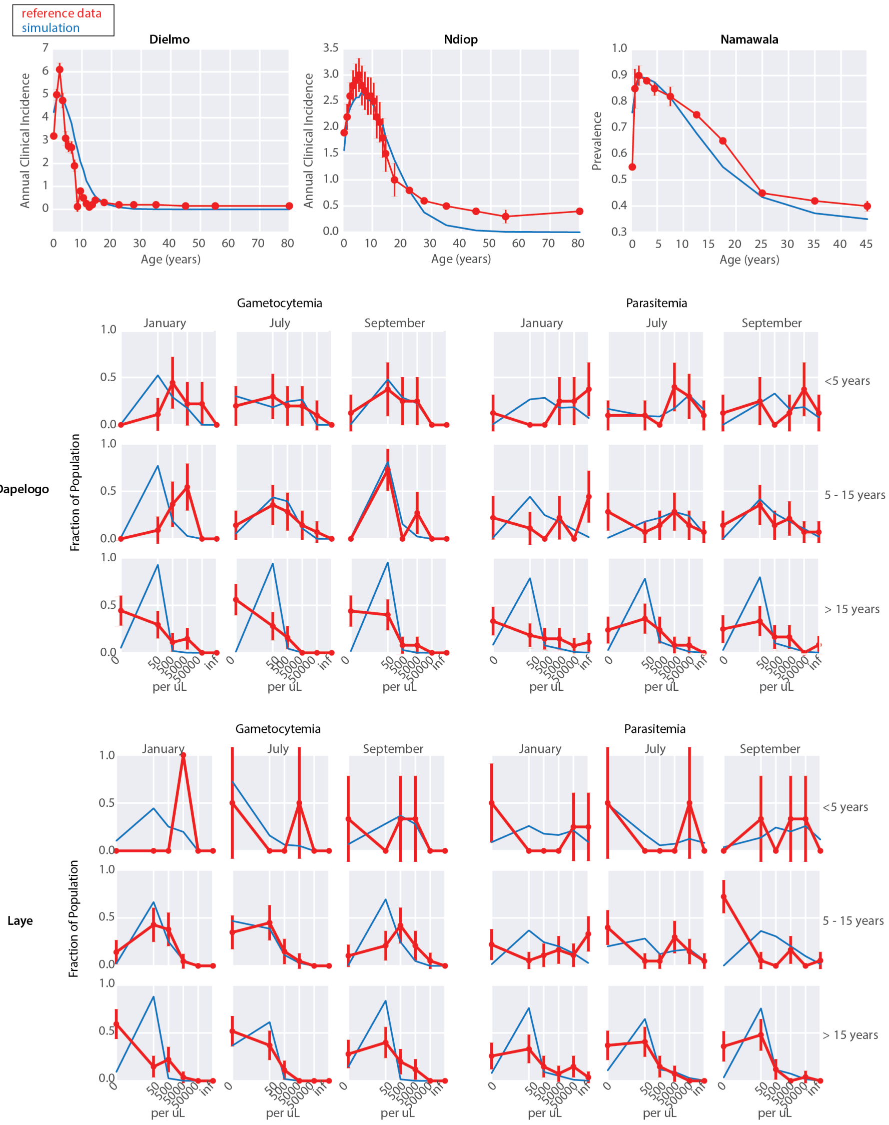

Figure 2: Comparison of reference data and calibrated simulation for Dielmo, Ndiop, Namawala, Dapelogo, and Laye study sites.

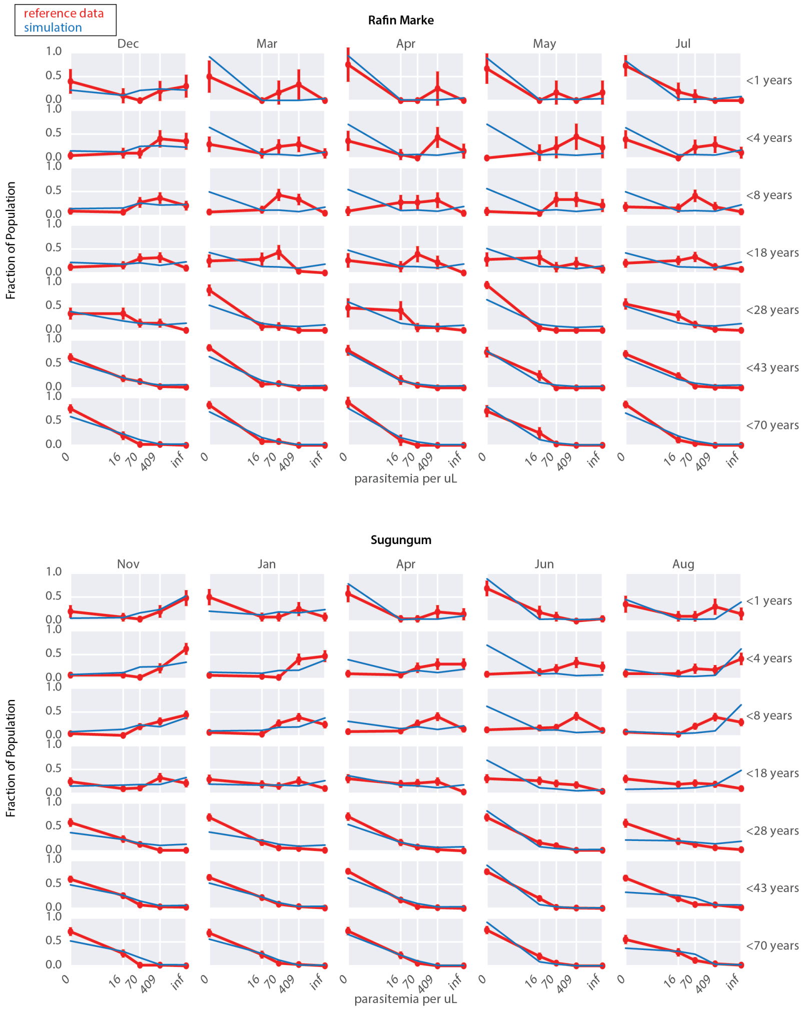

Figure 3: Comparison of reference data and calibrated simulation for Garki study sites not shown in main text.

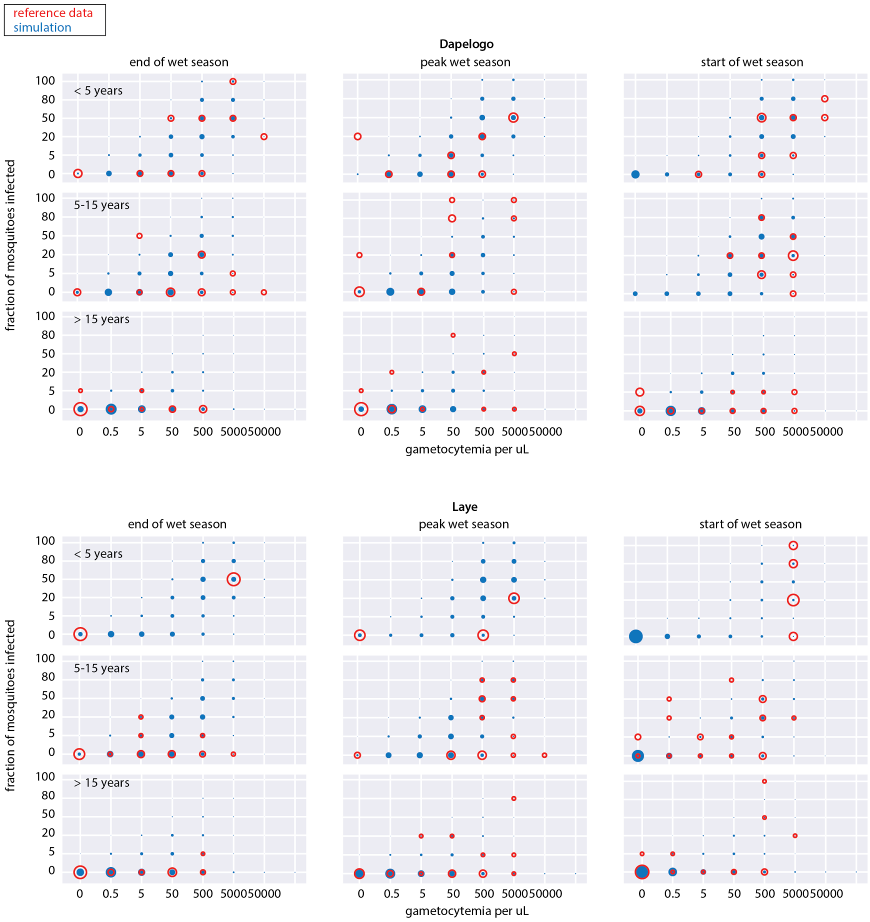

Figure 4: Comparison of reference data and calibrated simulation for relationship between gametocyte density and infectiousness to mosquitoes as measured in two Burkina Faso study sites.

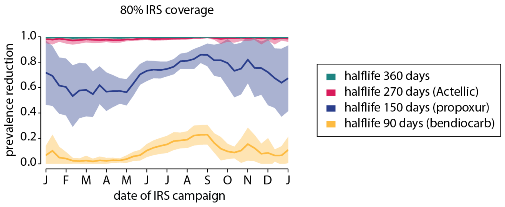

Figure 5: Optimal timing of IRS campaigns with IRS at 80% coverage.

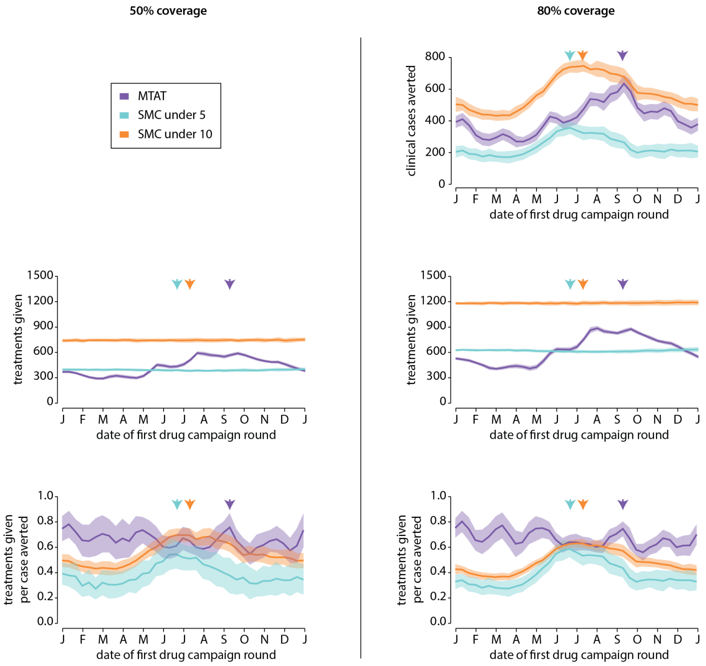

Figure 6: Cases averted and number of treatments given under various conditions of MTAT and SMC timing at 50 and 80% campaign coverage.

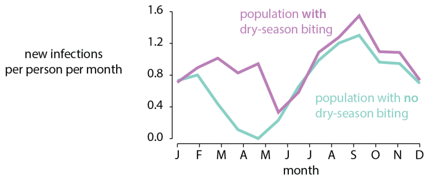

Figure 7: New infections per person per month in a population where 50% of individuals experience dry-season biting and 50% do not.
